# Reverse Genetic Approaches for the Generation of Full Length and Subgenomic Replicon of EV71 Virus

**DOI:** 10.3389/fmicb.2021.665879

**Published:** 2021-05-20

**Authors:** Hang Yang, Xiaohui Zhao, Meng Xun, Chaofeng Ma, Hongliang Wang

**Affiliations:** ^1^Department of Pathogen Biology and Immunology, Xi’an Jiaotong University Health Science Center, Xi’an, China; ^2^Department of Viral Diseases Laboratory, Xi’an Center for Disease Control and Prevention, Xi’an, China; ^3^Key Laboratory of Environment and Genes Related to Diseases, Xi’an Jiaotong University, Xi’an, China

**Keywords:** enterovirus (EV) 71, reporter virus, luciferase, replicon, drug screening

## Abstract

Enterovirus 71 (EV71) is a neurotropic pathogen that causes hand, foot, and mouth disease (HFMD) and it has been consistently associated with severe neurological, cardiac, and respiratory complications. Yet there is no specific treatment for this virus and we still know little about the viral pathogenesis. In this study, we first generated an infectious cDNA clone of EV71 virus from a patient virus strain and made a full-length virus with a NanoLuc reporter gene through reverse genetic approaches. The reporter gene of this virus is genetically stable when passaging in cells and could be used for antiviral testing. In addition, we also made subgenomic replicons (SGRs) of EV71, which lacks part of the structural genes dispensable for viral replication and showed that SGR can be used for viral replication study. Overall, these reporter viral systems are useful tools for EV71 pathogenesis study and antiviral screening.

## Introduction

In the past few decades, hand, foot, and mouth disease (HFMD) has broken out and spread in many parts of the world, especially in the Asia-Pacific region (Solomon et al., 2010). This disease mainly infects infants and children under 5 years old through the fecal-oral route. HFMD is generally considered as a self-limiting disease, with most patients recover within a week (Nassef et al., 2015). But a small number of patients can develop into severe symptoms, such as meningitis, poliomyelitis-like acute flaccid paralysis, brainstem encephalitis, and pulmonary edema (Ooi et al., 2010; Nassef et al., 2015; Aswathyraj et al., 2016). This disease is caused by various strains of enterovirus type A, most commonly enterovirus 71 (EV71), Coxsackievirus A16 (CV-A16) and CV-A6, with EV71 as the most frequently identified serotype among sever and fatal cases (Nassef et al., 2015; Li et al., 2020). There is no specific treatment for this disease and the reoccurrence of outbreaks underscores the importance of understanding the pathogenesis of these viruses and urgency for antiviral development.

**FIGURE 1:**
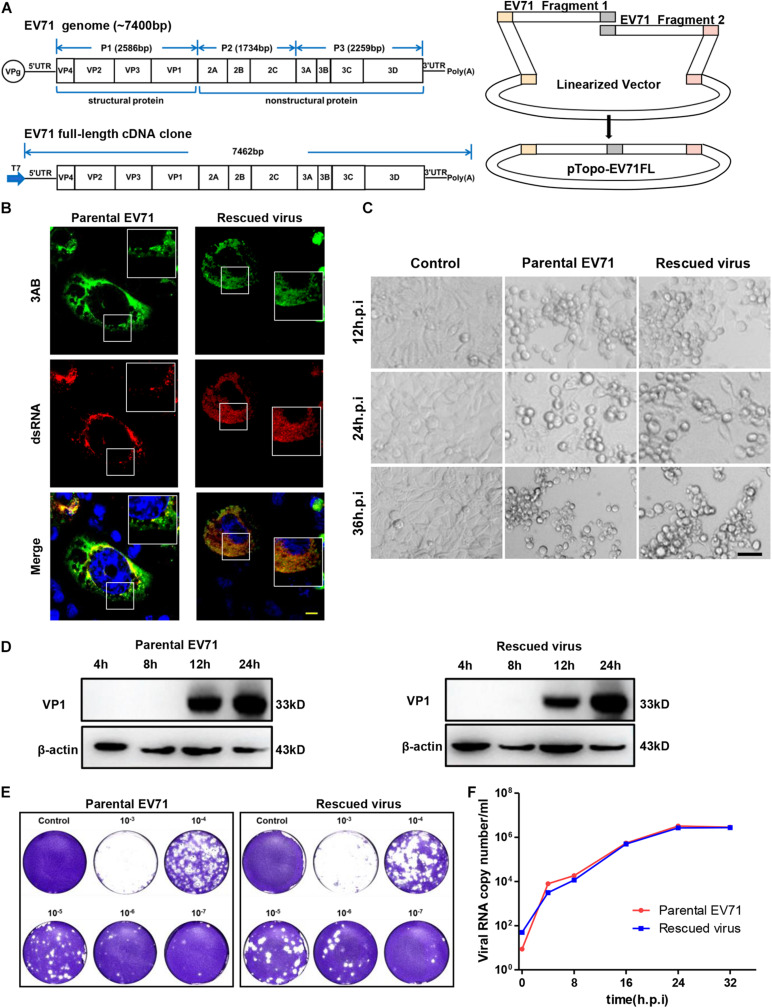
Characterization of rescued EV71 infectious clone. **(A)** Diagrams showing the genome of EV71 and cloning strategy used to make full-length infectious clone. **(B)** RD cells infected with parental or rescued EV71 virus were immunostained with anti-3AB and anti-dsRNA antibody. Nuclei were counterstained by DAPI. Bar, 10 μm. **(C)** Uninfected or RD cells infected with parental or rescued EV71 virus were observed at indicated time points for cytopathic effect. Bar, 100 μm. **(D)** RD cells infected with parental or rescued EV71 virus were immunoblotted with anti-VP1 antibody. β-actin was used as a loading control. **(E)** RD cells infected with serial dilutions of parental or rescued EV71 virus were analyzed by plaque assay. **(F)** Viral RNAs from RD cells infected with parental or rescued EV71 virus were quantitated by q-RT-PCR at various time points to compare the replication kinetics of these two viruses.

EV71 is a non-enveloped virus with a single-stranded, positive sense RNA genome of about 7.4 kb long. The viral genome consists of 5′ untranslated region (UTR), one open reading frame (ORF) and 3′ UTR (Figure 1A). The viral encoded protein-VPg protein is covalently attached to the 5′ UTR and the 3′ UTR has a poly(A) tail (Baggen et al., 2018). The single ORF is translated into one polyprotein in host cells which will then be cleaved into three precursor proteins: P1, P2 and P3. The precursor protein P1 can be further processed into 4 capsid proteins: VP1, VP2, VP3 and VP4; P2 and P3 can be cleaved to generate 7 non-structural proteins: 2A, 2B, 2C, 3A, 3B, 3C, and 3D (Liu et al., 2013; Wang and Li, 2019).

All viruses are obligate pathogens that depend on host cell machinery for replication. The complete EV71 life cycle includes viral entry, protein translation, viral RNA replication, particle assembly and release (Baggen et al., 2018). Recent efforts have greatly increased our knowledge of the infection of EV71 virus, but we are still far from understanding this virus. In addition, all the essential viral life cycle steps are potential targets for antiviral research (Baggen et al., 2018), but traditional antiviral screening methods either by monitoring cytopathic effect, or measurement of viral RNA by qPCR, are time-consuming and labor intensive. Therefore, reporter viruses are essential tools for viral pathogenesis study and high-throughput drug screening. Previous studies have reported several EV71 reporter viruses (Shang et al., 2013; Xu et al., 2015; Caine and Osorio, 2017), but those systems all have some disadvantages that limit their uses in infection study or drug screening.

Here, we constructed an infectious cDNA clone of EV71 from patient-isolated virus and used this as template to construct full-length and subgenomic replicon (SGR) reporter viruses with reverse genetics. We tested two different kinds of antivirals with our full-length reporter virus and found that this system could be adapted for high-throughput antiviral screening. In addition, the SGRs we generated has robust luminescence and are useful tools for virus replication and virus-host interaction studies.

## Results

### Construction of a Full Length Infectious Clone of EV71

The full-length EV71 cDNA clone, pTopo-EV71 was assembled by overlapping two fragments together with the pTopo backbone as depicted in Figure 1A and described in “Materials and Methods.” A T7 promoter was inserted upstream of EV71-5′ UTR for *in vitro* transcription. The *in vitro* transcribed RNA was transfected into RD cells and cytopathic effect (CPE) began to be observed after 12 h post-transfection (Supplementary Figure 1A). To confirm the cytotoxicity was caused by viral infection, viral VP1 expression was detected (Supplementary Figure 1B) by immunoblotting. All these suggested this rescued virus could successfully replicate in cells. Immunostaining was then conducted to stain the viral protein 3AB and dsRNA intermediates. Figure 1B showed that both parental and rescued virus showed colocalization of dsRNA and NS3AB, indicating the formation of replication complex within cells. Cell culture supernatant from viral RNA transfected cells was collected and used to infect naïve RD cells. Strong CPE was observed after 24 h post re-infection, suggesting the completion of viral life cycle in cells and successful release of infectious viral particles into supernatants. Virus stocks were generated after three times of passages and used for following experiments.

We next compared the rescued virus with parental virus in terms of viral protein expression, viral replication and ability to cause CPE. We found CPE started to appear around 12 h post-infection (h.p.i) and by 24 or 36 h.p.i, massive cell death was observed for both parental and rescued virus (Figure 1C), suggesting both viruses have comparable ability to cause cell death. Immunoblotting showed that VP1 protein expression was first detected at 12 h.p.i and continually to increase until 24 h.p.i (Figure 1D). To compare the virus titers of parental and rescued virus, a plaque formation assay was conducted. We found that both viruses reached virus titers over 10^7^ PFU/ml, suggesting similar replication abilities. In addition, the plaque morphology was also comparable between the two viruses, although the parental virus generated slightly smaller plaque in higher dilution (Figure 1E). Replication kinetics were also assayed with quantitative RT-PCR by measuring the RNA copy number at different time points, and as shown in Figure 1F, both viruses reached similar peak titers at around 32 h post-infection, suggesting they have similar replication kinetics. Overall, these data demonstrated that the rescued virus had comparable ability to replicate, release and cause CPE with parental virus.

### Construction of Infectious Clones Containing Reporter Gene

**FIGURE 2:**
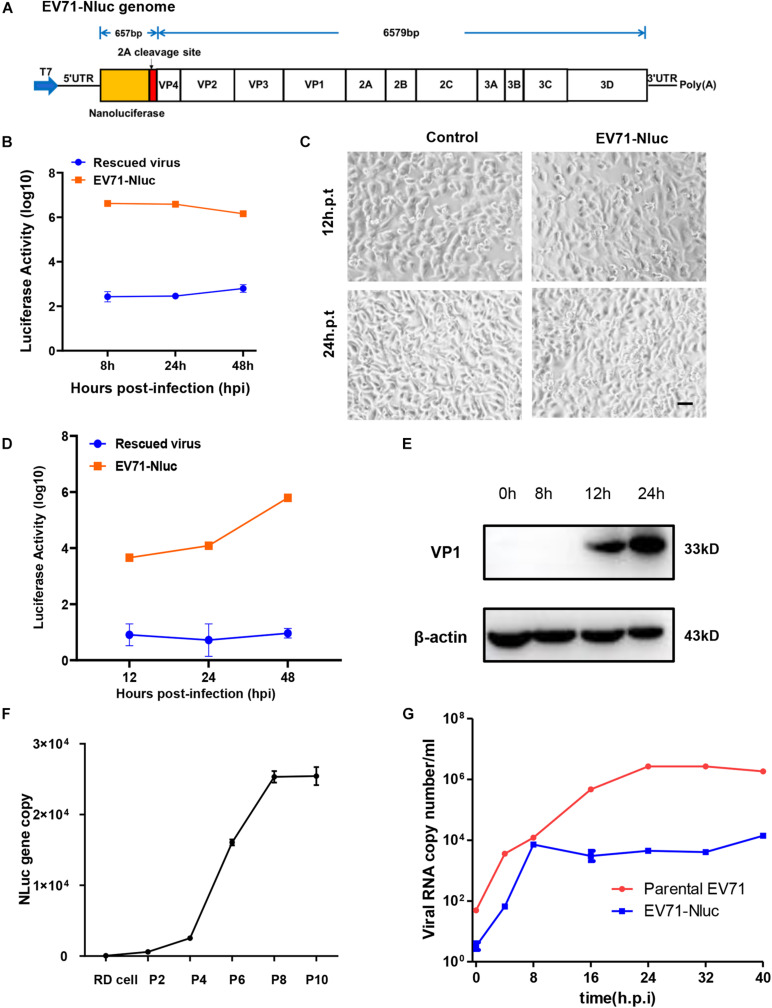
Characterization of full-length EV71 reporter virus. **(A)** Diagram showing the structure of the full-length EV71 reporter virus. **(B)** *In vitro* transcribed viral RNA with or without NanoLuc reporter gene was transfected into RD cells and luminescence was analyzed at different time points. **(C)** RD cells transfected with vehicle control or full-length EV71 reporter virus RNA were observed at indicated time points for cytopathic effect. Bar, 100 μm. **(D)** RD cells infected with EV71 reporter virus were analyzed for luminescence at indicated time points. **(E)** RD cells infected with EV71 reporter virus were immunoblotted with anti-VP1 antibody. β-actin was used as a loading control. **(F)** q-RT-PCR analysis of NanoLuc gene in RD cells infected with different passages of reporter virus. **(G)** Viral RNAs from RD cells infected with parental or reporter EV71 virus were quantitated by q-RT-PCR at various time points.

Reporter viruses are useful tools to study virus infection and viral pathogenesis. We next aimed to generate a full-length, infectious EV71 virus with a luciferase reporter. A NanoLuc luciferase reporter gene was inserted between EV71 5′ UTR and the start codon of VP4, and the EV71 2A cleavage site (ITTLG) was introduced after the reporter gene for efficient release of luciferase (Figure 2A). The luciferase activity was monitored after transfection of *in vitro* transcribed RNA into RD cells. And we found that at about 8 h post-transfection, NanoLuc signal was detected and kept active until 48 h post-transfection, while the rescued virus without luciferase reporter gene only had background reading (Figure 2B). However, compared to rescued EV71 virus (Supplementary Figure 1A), this reporter virus only caused slight CPE when transfected into RD cells (Figure 2C). To ensure the reporter virus can successfully fulfill its life cycle and be released into supernatant, we passaged the virus five times by inoculating supernatant into naïve cells. The NanoLuc signal was increasing over passages of virus stocks (Supplementary Figure 2A), suggesting the successful generation of full-length reporter virus and also indicating that the virus might get some adaptive mutations to enhance its propagation.

We next characterized the infection of this reporter virus. After infection, luciferase was detected about 12 h.p.i and kept climbing until 48 h.p.i (Figure 2D), suggesting the robust replication of this virus in cells. Immunoblotting showed that VP1 expression was detected 12 h.p.i and continually to increase until 24 h.p.i (Figure 2E), similar to those observed in the parental virus and rescued virus. Previously reports have shown that luciferase reporter gene inserted in the full-length EV71 genome can easily get lost during passaging (Shang et al., 2013). To make sure the NanoLuc reporter gene was stably maintained during the passages, viral RNA was extracted from different passages of virus stock, and the presence of NanoLuc gene was determined by qPCR. We found that NanoLuc gene was present in all passages of viruses tested (Figure 2F and Supplementary Figure 2B), suggesting this reporter virus was stable during passaging.

Since this virus has reduced CPE compared to parental virus, we tested viral RNA copy numbers at different time points and found that the replication of this reporter virus was attenuated relative to parental virus, but can still reach ∼10^4^ copy number/ml (Figure 2G). These results suggested that this reporter virus had slower viral replication kinetics.

### Reporter Virus for Antiviral Testing

**FIGURE 3:**
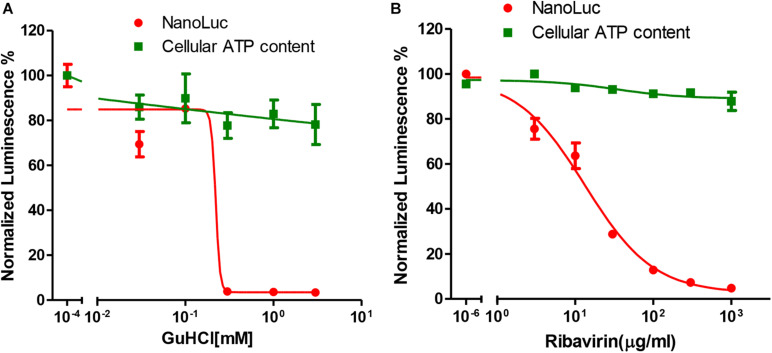
Antiviral tests of GuHCl and Ribavirin with Nanoluc reporter virus. **(A)** RD cells infected with full-length EV71 reporter virus were treated with indicated concentrations of GuHCl and NanoLuc luciferase was determined 24 h post-infection. Cellular ATP content was determined with Cell Titer Glo to indicate the toxicity of this compound. **(B)** RD cells infected with full-length EV71 reporter virus were treated with indicated concentrations of Ribavirin and NanoLuc luciferase was determined 24 h post-infection. Cellular ATP content was determined with Cell Titer Glo to indicate the toxicity of this compound.

NanoLuc luciferase is especially useful for drug screening due to its ability to be adapted to a homogenous format and the longer half-life of the luminescence with Nano-Glo assay system (Hall et al., 2012). We then tested whether this reporter virus could be used for antiviral assay. For this purpose, we tested two categories of chemicals that targeting different viral genes. GuHCl is an inhibitor of enterovirus targeting 2C helicase, while ribavirin is an inhibitor targeting 3D^pol^ protein. For both inhibitors, dose-responsive inhibition was observed for luciferase activity with an IC50 of 0.22 mM and 13.33 μg/ml respectively, while little cytotoxicity was observed (Figures 3A,B). These results suggested that this reporter virus could be used for various categories of EV71 antiviral inhibitor screening.

### Construction of Subgenomic Replicon of EV71

**FIGURE 4:**
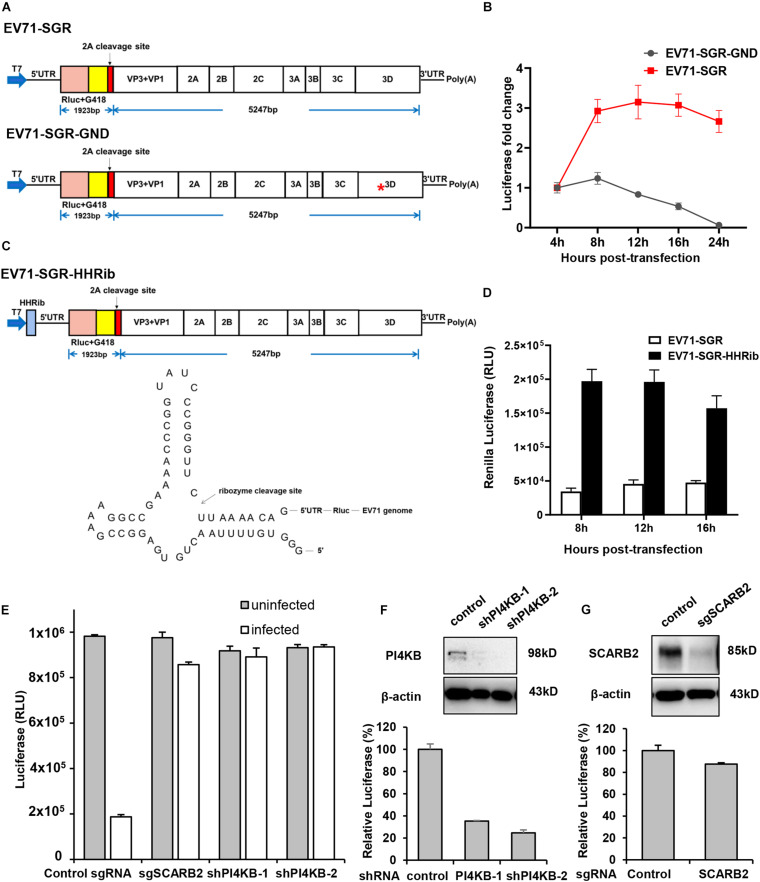
Characterization of EV71 subgenomic replicon. **(A)** Diagram showing the structure of the EV71 subgenomic replicons. Asterisk indicates the GND mutation in the polymerase catalytic site. **(B)** RD cells transfected with EV71 SGR or SGR GND mutant were assayed for luminescence at various time points. **(C)** Diagram showing the structure of the EV71-HHRib subgenomic replicons. The lower panel showed the RNA structure and the cleavage site of HHRib. **(D)** RD cells transfected with EV71 SGR or EV71-SGR-HHRib were assayed for luminescence at various time points. **(E)** RD cells transduced with control sgRNA or SCARB2 sgRNA, PI4KB shRNA lentivirus were either mock infected or infected with EV71 virus for 24 h and cell viability was determined with Cell Titer Glo. **(F)** RD cells transduced with control or PI4KB shRNA lentivirus were transfected with EV71 SGR RNA and luciferase activity was determined 12 h post-transfection. Cells described above were also immunoblotted with the indicated antibody to show the inhibition of PI4KB. **(G)** RD cells transduced with control or SCARB2 sgRNA lentivirus were transfected with EV71 SGR RNA and luciferase activity was determined 12 h post-transfection. Cells described above were immunoblotted with the indicated antibody to show the inhibition of SCARB2.

In addition to full-length reporter virus, subgenomic replicons (SGRs) are also powerful tools for viral studies, especially for viral replication studies. Therefore, we next generated a subgenomic replicon with a *Renilla* Luciferase/neomycin-resistant gene based on our full-length infectious cDNA clone. Structural genes are generally dispensable for viral replication and most virus SGRs comprise only the non-structural genes. However, a previous study had shown that part of the capsid coding sequence is required for efficient replication of human rhinovirus 14 (McKnight and Lemon, 1996), another member of the Enterovirus genus in the family of Picornaviridae. Therefore, we generated our subgenomic replicon by replacing the VP4, VP2 and part of VP3 with *Renilla* Luciferase reporter gene followed by the EV71 2A cleavage site (Figure 4A). Replication of this replicon was assessed in RD cells and luciferase activity was determined at various time points to indicate the replication of this replicon. From Figure 4B, we can see that the luciferase activity of EV71 SGR increased substantially from 4 to 16 h post-transfection, while the luciferase of SGR with a catalytic site GND mutation failed to increase, suggesting our subgenomic EV71 replicon replicated efficiently.

In addition, it has been reported that a precise 5′ end is required for efficient viral RNA replication for a number of positive—and negative-sense RNA viruses and the introduction of hammerhead ribozyme (HHRib) would generate viral RNAs with authentic 5′ end (Herold and Andino, 2000; Wang et al., 2020). For this purpose, we inserted a cDNA copy of -HHRib—downstream of T7 promoter to ensure a precise 5′ end could be generated after transcription (Figure 4C). Indeed, the replication of subgenomic RNA increased significantly due to removal of extra sequences by HHRib (Figure 4D), suggesting a precise 5′ end can enhance viral RNA replication.

Stable cell lines harboring the SGRs are useful tools for screening antiviral inhibitors targeting viral replication or translation steps, we therefore tried to establish stable cell lines containing the EV71 SGR. We transfected RD cells with different SGRs (SGR with VP4-VP1 deletion; SGR with VP4, VP2 and part of VP3 deletion and SGR with part of VP2 and VP3 deletion, Supplementary Figure 3A), however, G418 selection led to no survival cells even after repeated attempts. It has been reported that G418 resistance occurred at a very low frequency in HCV SGR transfected cells and bicistronic replicon with encephalomyocarditis virus (EMCV) IRES was able to enhance viral replication capability (Blight et al., 2000; Bartenschlager, 2002). We also employed this strategy and designed a bicistron replicon (Supplementary Figure 3B). Transient viral replication was observed after transfection but luciferase signal went down after 12 h (Supplementary Figure 3C). G418 selection of RD or Vero cells transfected with this replicon RNA also failed to generate stable cell lines.

Due to the lack of structural genes, SGRs enables uncoupling of viral replication and translation from virus entry and assembly, and therefore, they are especially useful in viral replication studies. Scavenger receptor B2 (SCARB2) is a viral receptor for EV71 (Yamayoshi et al., 2009; Yamayoshi et al., 2012) while phosphatidylinositol-4-kinase IIIβ (PI4KB) is an essential host factor for enterovirus replication organelle formation and viral replication (Hsu et al., 2010; Xiao et al., 2017). Inhibition of either SCARB2 or PI4KB led to significant decrease of EV71 infection in RD cells (Figure 4E), indicating both host factors are required for EV71 propagation. However, when EV71 SGR RNA was transfected into these specific host factor-deficient cells, only PI4KB inhibition led to decrease of viral RNA replication (Figure 4F), while SCARB2 knockout had little effect (Figure 4G), consistent with the fact that PI4KB plays its roles during viral replication and SCARB2 functions during viral entry. These results highlight the importance of SGRs in viral replication studies.

## Discussion

In this study we established EV71 full-length reporter virus and subgenomic replicon systems with reverse genetics, which are useful tools for viral pathogenesis studies as well as antiviral screening. We did not observe any difference between the parental EV71 and the rescued infectious clone in terms of replication dynamics, viral cytopathic effects on RD cells and plaque morphologies, suggesting the latter could fully recapitulate the properties of the wild-type parental strain. However, the full-length virus with luciferase reporter showed attenuated replication kinetics and lower CPE, and this is in line with previous reports that insertion of eGFP or *Gaussia* luciferase into EV71 genome both impaired viral replication (Shang et al., 2013; Xu et al., 2015). And this seems to be true for many other viruses, like Hepatitis C virus (Moradpour et al., 2004; Wang and Tai, 2017), Dengue virus (Schoggins et al., 2012; Suphatrakul et al., 2018), Zika virus (Shan et al., 2016; Münster et al., 2018), most probably due to the size selection of viral genome during viral replication and encapsidation.

Previous studies have reported the generation of full-length EV71 viruses with either eGFP or *Gaussia* luciferase reporter gene (Shang et al., 2013; Xu et al., 2015). Although these reporter viruses are useful tools for viral pathogenesis study and can be used for antiviral testing, they have their limits. For example, GFP reporter virus has disadvantages when used for high-throughput antiviral screening in terms of sensitivity and ease of use. While *Gaussia* luciferase is sensitive, its long half-life and high background also limit its uses in high-throughput screening. Here, we generated a Nanoluc based full-length reporter virus, which gives high sensitivity and low background. In addition, the luciferase we used in this study has a short half-life due to the introduction of PEST (Hall et al., 2012), and can be tested in homogenous system, which make this reporter virus especially suitable for antiviral screening. Since this is a full length reporter virus, it can be used for screening of antivirals targeting any steps of viral life cycle. Here we tested two different kinds of EV71 inhibitors, one targeting viral translation, the other inhibiting viral replication, both of which showed good dose-response inhibition. In addition, this assay could be easily scaled down to 384- or 1,536-well format, therefore, it is especially useful for high-throughput antiviral screening.

In addition to the full-length reporter virus, we also made SGRs with luciferase reporter gene. Previous reports of EV71 SGR are to simply replace viral structural gene region (VP4-VP1) with a luciferase reporter (Chen et al., 2012; Kim et al., 2016). As part of the structural genes are essential for picornavirus RNA replication (McKnight and Lemon, 1996), we included part of structural genes in the SGRs, and get more sustained replication compared to a previous report (Kim et al., 2016). In addition, with the introduction of HHRib, which produces viral RNA of precise 5′ end, this replicon gave increased luciferase signal than the one with no HHRib, suggesting robust replication of this replicon in cells. As some structural genes are missing in the SGR, this viral RNA could not produce infectious progeny in the supernatant. Therefore, SGRs improve the safety of viral handling and they also uncouple viral replication, translation from virus entry and assembly, and thus they are useful tools to study host factor roles in viral replication. In this study, we tested two host factors involved in viral entry and replication, respectively, and found that only PI4KB could affect the luciferase signal of this SGR, suggesting this system can be used to decipher the roles of host factors in viral replication.

Although these SGRs could also be used for screening antiviral inhibitors targeting viral replication or translation (Kim et al., 2016), it is time-consuming and of high variability because of the transfection step. Therefore, stably cell lines harboring SGR could be an ideal alternative for this purpose. However, stable cell lines harboring EV71 SGR were not successful in our hands even with EMCV IRES to enhance viral RNA replication, suggesting some adaptive mutations are required for efficient replication. Indeed, stable cell lines harboring SGR are successful only in several viruses, mainly in the flaviviridae family, like Hepatitis C virus (Lohmann et al., 1999; Bartenschlager, 2002), Dengue virus (Ng et al., 2007), etc., and most of them are associated with adaptive mutations (Bartenschlager, 2002; Khan et al., 2020).

In summary, here we generated full-length and subgenomic replicon with reporter genes for EV71 virus, these systems will facility studies concerning viral gene functions, viral pathogenesis as well as antiviral screening.

## Materials and Methods

### Cell Lines, Virus, and Reagents

Human rhabdomyosarcoma (RD) cells were maintained in Dulbecco’s Modified Eagle Medium (DMEM; Gibco) supplemented with 10% fetal bovine serum (FBS), 100 U/ml penicillin, 1 μg/ml streptomycin at 37°C in presence of 5% CO_2_. The EV71 strain was isolated from patient at Xi’an municipal CDC, propagated and expanded in RD cells. This viral stock was used as parental virus. GuHCl was purchased from Sigma-Aldrich and ribavirin was from MedChemExpress. EV71 VP1 and 3AB antibody were purchased from GeneTex. dsRNA antibody (J2) was from English and Scientific Consulting, Hungary. PI4KB antibody was from Proteintech Group and SCARB2 antibody was from Santa Cruz Biotechnology. β-actin antibody was from ABclonal Technology. DAPI, Alexa Fluor conjugated secondary antibodies for microscopy experiments were purchased from Thermo Fisher Scientific.

### Plasmids Construction

The overall strategy for the full-length EV71 cDNA clone was depicted in Figure 1A. The viral RNA was extracted from virus infected RD cells with GeneJET RNA purification kit (Thermo Fisher Scientific) and reversed transcribed with random primer and RevertAid Reverse Transcription kit (Thermo Fisher Scientific). Two overlapping fragments (F1:1-3786; F2: 3707-7442) covering the entire EV71 genome was amplified with cDNA as template. A T7 promoter sequence (TAATACGACTCACTATAGG) was included upstream of 5′ UTR region. The primers used are listed in Table 1. These two fragments as well as a linearized vector pTopo were assembled with NEBuilder^®^ HiFi DNA Assembly Master Mix according to manufacturer’s instruction and transformed into Stable3 competent cells (AlpaLife). The colonies are screened by colony PCR and then confirmed with sequencing. The correct clone was named as pTopo-EV71FL.

**TABLE 1:** Primers used for DNA construct preparation.

**Construct**	**Fragment**	**Forward/Reverse**	**Sequence**
pTopo- EV71FL	F1	F	TAATACGACTCACTATAGGTTAAAACAGCCTGTGGGTTGCACCCACT
		R	GACACACCCTGTTCCATAGC
	F2	F	GTGGCAATGGGCTCGTTGG
		R	CTAGAAGCTTTTTTTTTTTTTTTTTTTTTTTTTTTTTTTTTTTTTTTGCTATTCTGGTTATAACA AATTTAC
pTopo- EV71FL	F1	F	CAACGATCGGAGGACCG
		R	ccaaGGGTAGTAATGGCACGCGTGCCCATGTTTAGCTGTGTTAA GGGTCAAG
	F2	F	ATGGGCACGCGTGCCATTACTACCCTTGGTTCGCAAGTGTCTACACAGCGCTCCGG
		R	GCCGGTAGCC ATGAAGGATC CAGTAAACAT
pTopo- EV71FL-NL		F	ctagACGCGT ATGGTCTTCACACTCGAAG
		R	AgtcACGCGT GACGTTGATGCGAGCTGAAG
pTopo- EV71SGR-RL		F	Agct ACGCGT ATGGCTTCCAAGGTGTACG
		R	TGAAGGATCCAAGGGTAGTAATGGCGAAGAACTCGTCAAG AAGGCG
pTopo- EV71SGR-GND	F1	F	TGAACAGGCCCTGTTCTC
		R	TAGCGAGCAC ATCGTTTCCA TAAGCAACCA TGTTGAGTTC ATC
	F2	F	ACATGGTTGCTTATGGAaACGATGTGCTCGCTAGTTATCCC
		R	CAGGAAACAGCTATGAC
pTopo- EV71SGR-HHRib	F1	F	CAACGATCGGAGGACCG
		R	CTAGATAATACGACTCACTATAGGGGGTGTTTTAACTGATGAGGCCGAAAGGCCGAA AA
	F2	F	CAACCCACAGGCTGTTTTAAGAACCCGGGATACCGGGTTTTCGGCCTTTCGGCCTCATC
		R	CCATACGCGTcgagcc

A two-step strategy was employed to get the full-length reporter virus cDNA. First, a *Mlu*I restriction site as well as the EV71 2A cleavage site (ITTLG) coding sequence were first introduced between 5′ UTR and VP4 coding region of pTopo-EV71FL by overlap extension PCR. Then the NanoLuc-PEST encoding gene was amplified form pNL1.2 vector (Promega Corporation) and inserted within this *Mlu*I site. The primers used are listed in Table 1. The construct was confirmed by sequencing and named as pTopo-EV71FL-NL.

To get the subgenomic replicon cDNA, the pTopo-EV71-NL was first digested with *Mlu*I and *Bam*HI to remove the VP4-VP2-VP3(part) region. A *Renilla* luciferase (Rluc)/Neomycin-resistance encoding gene amplified from pSGR-JFH1(NS5A/SNAP) (Wang and Tai, 2017) as well as the 2A cleavage site encoding sequence were ligated with this backbone. The correct construct was named as pTopo-EV71SGR-RL. To generate the polymerase-defective mutant of SGR EV71, the catalytic site of polymerase was mutated to GND by overlap extension PCR. The HHRib coding sequence (GGGTGTTTTAACTGATGAGGCCGAAAGGCCGAAAACCC GGTATCCCGGGTTCTTAAAACAG) was inserted after the T7 promoter to make the pTopo-EV71SGR-HHRib construct. The primers used are listed in Table 1. The complete genome sequences of the above constructs are available upon request.

### *In vitro* Transcription and RNA Transfection

To generate viral RNA, constructs were first linearized with *Hin*dIII and *in vitro* transcribed with MEGAscript^TM^ T7 Transcription Kit (Thermo Fisher Scientific). The RNA was subjected to DNase digestion and purified with GeneJET RNA column purification prior to transfection into RD cells using TransIT-mRNA transfection reagent (Mirus Bio) according to manufacturer’s instruction.

### Plaque Assay

To determine the virus virulence of parental EV71 virus and rescued virus, viral supernatants were collected and adjusted to the same MOI. Tenfold serial dilutions of virus were then added to RD cells grown in 6-well plate and incubated for 1 h at 37°C. Then inoculums were then removed and cells were cultured with DMEM containing 2% FBS and 1% low melting point agarose (LMPA). Three days later, infected cells were fixed with 4% paraformaldehyde and viral plaques were stained with 1% crystal violet.

### Viral RNA Quantification

RD cells in 12-well plate were infected with same MOI of virus and harvested at various time points. Total RNAs were extracted with GeneJET RNA Purification Kit and then reversed transcribed as described above. Quantification of viral RNA was performed by real-time quantitative PCR with SYBR Mix (Hieff^TM^ qPCR SYBR^TM^ Green Master Mix) using specific primers listed in Table 2. pTopo-EV71FL-NL plasmid was serially diluted to generate a standard curve.

**TABLE 2:** Primers used for qPCR.

**Target**	**Forward/Reverse**	**Sequence**
VP1 qPCR	F	AAGGTTCCAGCACTCCAAGC
	R	TCTCCAACTAATCCCGCCC
GAPDH qPCR	F	CTCTGCTCCTCCTGTTCGAC
	R	TTAAAAGCAGCCCTGGTGAC
NanoLuc qPCR	F	GATTGTCCTGAGCGGTGAAA
	R	CATACGGCCGTCCGAAATAG

### Immunofluorescence

Immunofluorescence staining was carried out as previously describe (Wang and Tai, 2017). Briefly, cells were seeded on coverslips coated with Poly-D-lysine 1 day before they were infected with EV71 virus (MOI 0.01) and incubated for another 24 h. Cells were then fixed with ice-cold methanol for 10 min, followed by 2% FBS block for 1 h. Cells were then stained with dsRNA antibody (J2, English and Scientific Consulting, Hungary) and EV71 3AB (GeneTex). The images were acquired with Nikon C2 confocal microscope.

### Western Blotting

Cells infected with virus were harvested at various time points and lysed with 1 × LDS-loading buffer (Thermo Fisher Scientific). SDS-PAGE were carried out followed by transferring to a PVDF membrane. The blots were then probed with indicated antibody. Following TBST wash, corresponding secondary antibodies were added and bands were visualized with SuperSignal^TM^ West Femto Maximum Sensitivity Substrate (Thermo Fisher Scientific).

### Luciferase Assay

RD cells transfected with EV71-FL-NL RNA or infected with Nanoluc reporter virus were harvested at indicated time points and luciferase activity was determined with Nano-Glo^®^ Luciferase Assay System (Promega Corporation) according to manufacturer’s instructions. Luciferase was measured with BioTek Neo2 microplate reader. For GuHCl or ribavirin inhibition tests, RD cells were first infected with reporter virus, 4 h later, cells were treated with indicated concentrations of inhibitor for another 24 h before luciferase were measured. Cell viability were determined with Cell Titer Glo (Promega Corporation) to measure the cellular ATP contents.

### shRNA Knockdown or CRISPR Knockout Cell Line Construction

shRNA or CRISPR lentiviruses pseudotyped with VSV-G protein were produced as previously described (Wang et al., 2014; Wang and Tai, 2019). Briefly, pLKO.1-Puro based shRNA or pLentiCRISPR V2 based sgRNA lentiviral vector and psPAX2, pMD2.G were co-transfected into 293T cells and supernatants were harvested 50 h later, filtered with 0.45 μm filter and used for transduction. sgRNA sequences and shRNA sequences used were listed in Table 3. Cells transduced with lentiviral particles were then selected with puromycin for stable cell lines.

**TABLE 3:** sgRNA and shRNA target sequences.

**Gene**	**Sequence**
sgSCARB2	TGTAGACCAGAGTATCGAGA
shPI4KB-1	CGACATGTTCAACTACTATAA
shPI4KB-2	GCAAGAAACACGAAGGATCAT

## Data Availability Statement

The original contributions presented in the study are included in the article/Supplementary Material, further inquiries can be directed to the corresponding author/s.

## Author Contributions

All authors contributed to the study design, data analysis, and manuscript preparation.

## Conflict of Interest

The authors declare that the research was conducted in the absence of any commercial or financial relationships that could be construed as a potential conflict of interest.
