# Supplementary material for: Reverse Genetic Approaches for the Generation of Full Length and Subgenomic Replicon of EV71 Virus
Source: Front Microbiol. 2021 May 20;12:665879. doi: 10.3389/fmicb.2021.665879 (PMC8172962; doi:10.3389/fmicb.2021.665879)
Supplement: Supplementary file 1 [file Image_1.pdf]

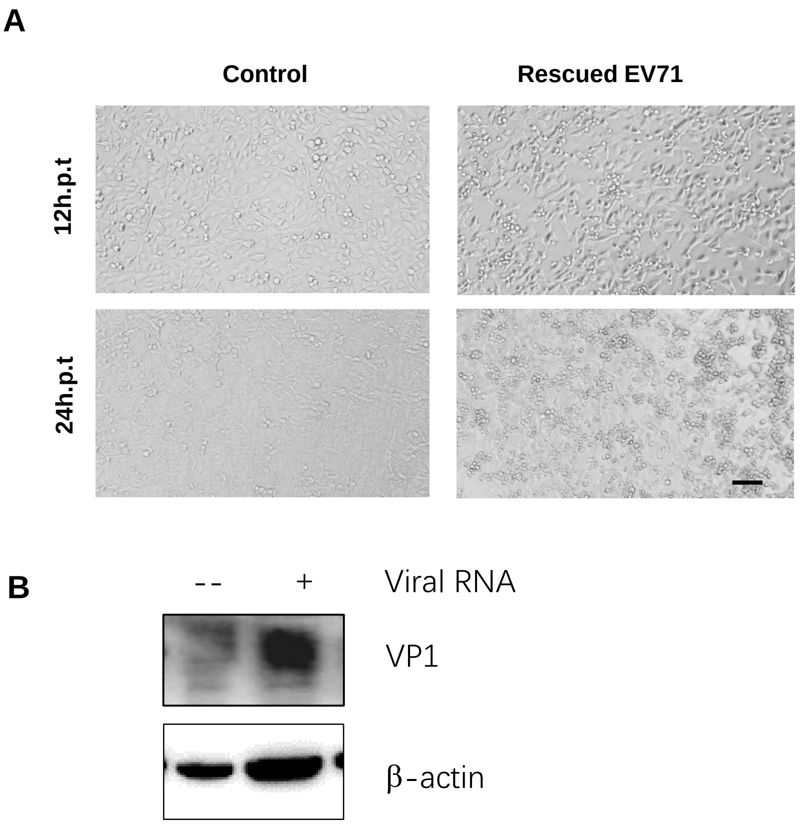

Supplement Figure 1

A. RD cells transfected with in vitro-transcribed EV71 viral RNA were observed at indicated time points for cytopathic effect. Bar, 100μm.
B. Control or in vitro-transcribed EV71 viral RNA were transfected into 293T cells and VP1 expression were determined by immunoblotting. β-actin was used as a loading control.

A

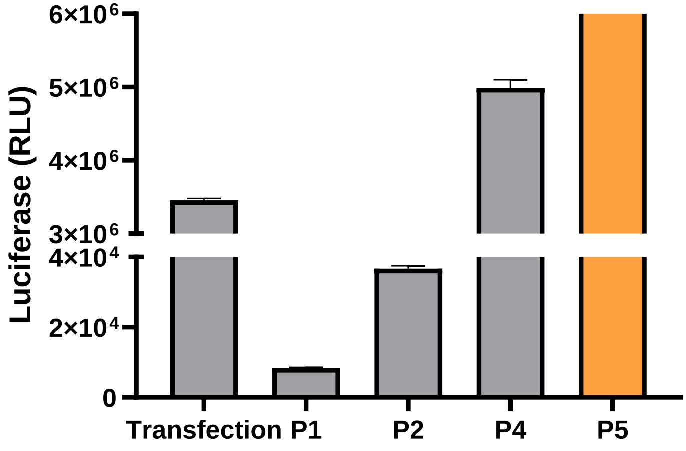

B

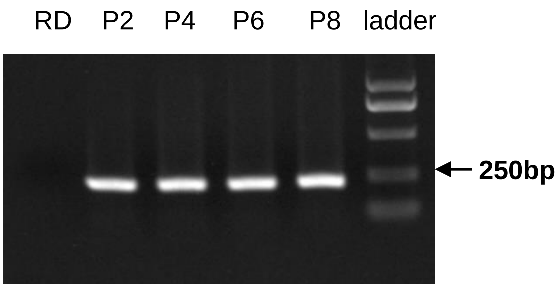

Supplement Figure 2

A. RD cells transfected with in vitro-transcribed reporter viral RNA or infected with different passages of reporter virus were determined for luciferase expression. NanoLuc signals increased with passages of virus. P5 virus showed signal exceeding the detection limit of Neo2 plate reader.
B. RT-PCR analysis of NanoLuc gene in RD cells infected with different passages of reporter virus.

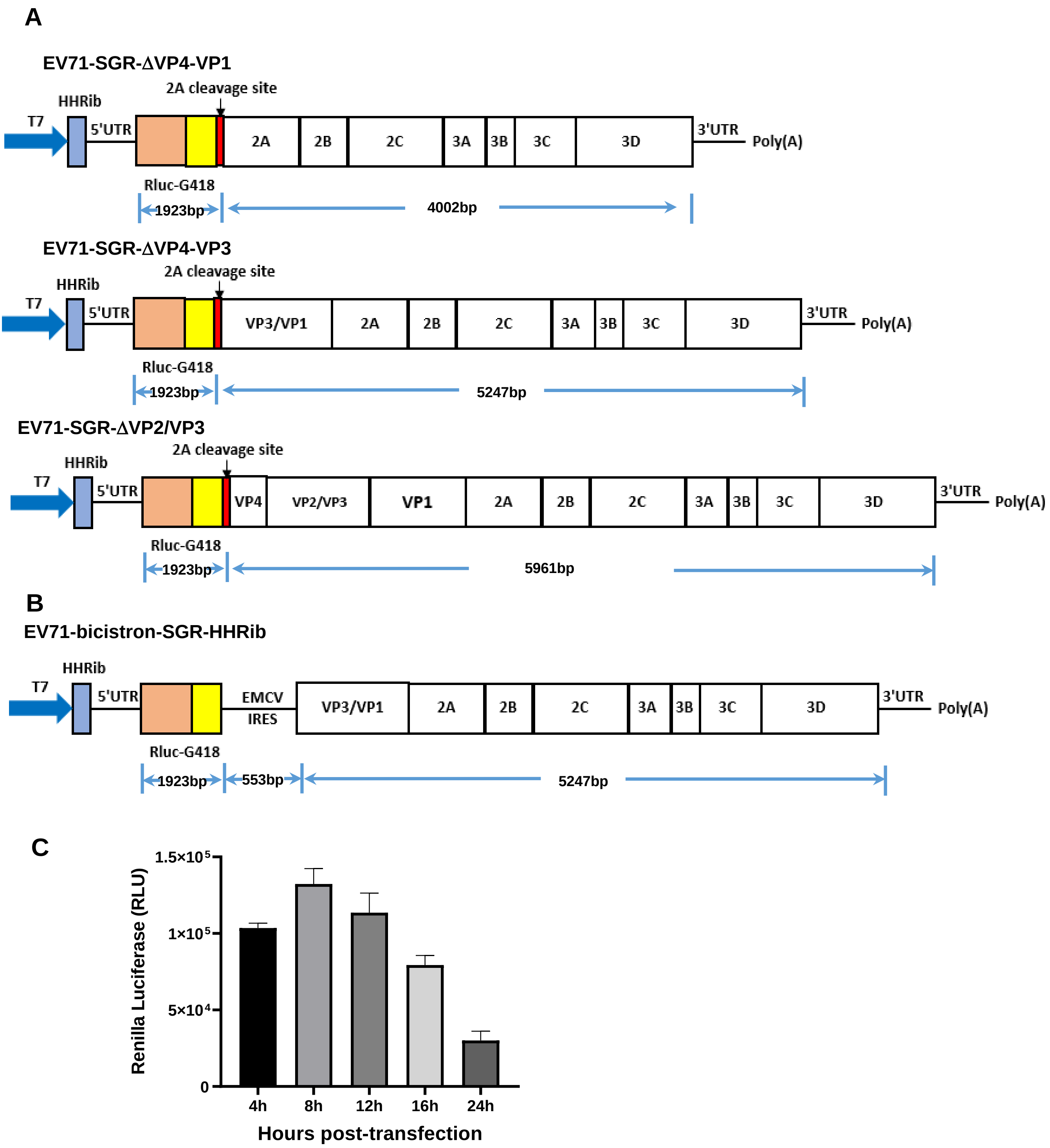

Supplement Figure 3

A. Diagram showing the structure of the EV71 subgenomic replicons.
B. Diagram showing the structure of a bicistronic EV71 subgenomic replicons, an EMCV IRES was inserted upstream of VP3 coding region.
C. RD cells transfected with the bicistronic SGR were assayed for luminescence at various time points.
